# An Effective LSTM Recurrent Network to Detect Arrhythmia on Imbalanced ECG Dataset

**DOI:** 10.1155/2019/6320651

**Published:** 2019-10-13

**Authors:** Junli Gao, Hongpo Zhang, Peng Lu, Zongmin Wang

**Affiliations:** ^1^Cooperative Innovation Center of Internet Healthcare, Zhengzhou University, Zhengzhou 450000, China; ^2^State Key Laboratory of Mathematical Engineering and Advanced Computing, Zhengzhou 450001, China; ^3^Department of Automation, School of Electrical Engineering, Zhengzhou University, Zhengzhou 450001, China

## Abstract

To reduce the high mortality rate from cardiovascular disease (CVD), the electrocardiogram (ECG) beat plays a significant role in computer-aided arrhythmia diagnosis systems. However, the complex variations and imbalance of ECG beats make this a challenging issue. Since ECG beat data exist in heavily imbalanced category, an effective long short-term memory (LSTM) recurrence network model with focal loss (FL) is proposed. For this purpose, the LSTM network can disentangle the timing features in complex ECG signals, while the FL is used to resolve the category imbalance by downweighting easily identified normal ECG examples. The advantages of the proposed network have been verified in the MIT-BIH arrhythmia database. Experimental results show that the LSTM network with FL achieved a reliable solution to the problem of imbalanced datasets in ECG beat classification and was not sensitive to quality of ECG signals. The proposed method can be deployed in telemedicine scenarios to assist cardiologists into more accurately and objectively diagnosing ECG signals.

## 1. Introduction

Cardiovascular diseases (CVDs) are the leading cause of death worldwide [1]. According to the World Health Organization, about 17.9 million people died of CVD in 2016, accounting for 31% of all deaths. Arrhythmia is caused by improper intracardiac conduction or pulse formation, which can affect heart shape or disrupt the heart rate [2]. An electrocardiogram (ECG) is a comprehensive manifestation of the electrical signal activity of the human heart. Obtaining the detailed physiological state of various parts of the heart by collecting signals is an indispensable means of clinical objective diagnosis. Automated analysis and diagnosis based on ECG data have a reliable clinical diagnostic reference value for arrhythmia [3].

Many methods for automatic classification of ECGs have been proposed. The type of ECG beat can be distinguished by the time-domain [4], wavelet transform [5], genetic algorithm [6], support vector machine (SVM) [7], Bayesian [8], or other methods. Although the above classification methods achieve high accuracy on experimental datasets, their performance is highly dependent on the extraction characteristics of fixed or manual design methods. Manually designing extracted features may increase computational complexity throughout the process, especially in the transform domain.

Deep learning constitutes the mainstream of machine learning and pattern recognition. It provides a structure in which feature extraction and classification are performed together [9]. Deep learning has been widely used in many fields, such as image classification [10], target detection [11], and disease prediction [12]. It is also effectively used to analyze bioinformatics signals [13–17]. Acharya et al. [13] proposed a nine-layer convolutional neural network (CNN) to automatically identify five ECG beat types. Yildirim et al. [15] designed an end-to-end 1D-convolutional neural network (1D-CNN) model for arrhythmia detection. Hannun et al. [16] developed a deep neural network (DNN) to detect 12 rhythm ECG classes. Oh et al. [17] used U-Net autoencoder to detect five arrhythmias. The input of CNN through its unique weight-sharing mechanism is a spatial change, that is, the spatial data with the image as a typical example perform well. However, recurrent neural networks (RNNs) are more appropriate for chronological changes in the appearance of sample sequences.

Long short-term memory (LSTM) network is a special type of RNN that is widely used for time series analysis. It can effectively retain historical information and realize learning of long-term dependence information of text. It has been used in many fields, such as natural language processing [18] and speech recognition [19]. LSTM is also used for the detection of ECG arrhythmias. [20–23]. Yildirim [20] proposed a new model for deep bidirectional LSTM network- (BLSTM-) based wavelet sequences (WS) to classified electrocardiogram (ECG) signals. Oh et al. [22] proposed a combined network model using CNN and LSTM for ECG arrhythmia diagnosis. Hou et al. [23] introduced a new algorithm based on deep learning that combines LSTM with SVM for ECG arrhythmia classification.

The imbalance of the ECG dataset is an additional challenge to accurately classify ECG beats. There are two problems in the training process: (1) low training efficiency, because normal ECG beats occupying a large proportion of the dataset are prone to negative effects, and (2) degeneration of the model when a normal ECG beat overwhelms training. Some researchers have attempted to address imbalance in the ECG beat data when diagnosing arrhythmia. Sanabila et al. [24] used the generated oversampling method (GenOMe) to solve the problem of imbalanced arrhythmias, which generated new data points with specific distributions (beta, gamma, and Gaussian) as constraints. Rajesh and Dhuli [25] employed three data-level preprocessing techniques on an extracted feature set to balance the distribution of ECG heartbeats. These were random oversampling and undersampling (ROU), synthetic minority oversampling technique with random undersampling (SMOTE + RU), and distribution-based balancing (DBB). As an alternative to resampling the input ECG beat data or feature set, focal loss addresses imbalanced dataset classification by downweighting easy normal ECG beat examples so that their contribution to the loss is small even if their number is large, that is, focal loss concentrates network training on hard ECG beat types, which may constitute a small part of the dataset.

Inspired by the idea of FL to solve the problem of imbalanced category classification and LSTM popularization technology, an effective LSTM with FL is proposed to handle imbalanced ECG beat data on the MIT-BIH arrhythmia database. LSTM automatically extracts the timing characteristics of complex ECG signals, and FL mitigates the problem of ECG class imbalanced distribution faced by the LSTM network, enabling the network to effectively train all categories. The experimental results show that the proposed model achieved state-of-the-art performance on imbalanced ECG beat data and outperformed previous results. Furthermore, we conduct experiments on both denoised and without denoised ECG datasets, and results demonstrate the proposed model is not sensitive to quality of ECG signals.

## 2. Methodology

Arrhythmia classification using deep learning generally includes two basic stages: preprocessing and classification. In the preprocessing stage, the Daubechies 6 (db6) discrete wavelet transform is used to remove noise from the ECG signal. The ECG heartbeat is then extracted using the sliding window search method, and the data are normalized using Z-score. The LSTM network is proposed for ECG heartbeat classification. The details and theoretical background of these methods are discussed in the following sections.

### 2.1. Preprocessing

Preprocessing includes denoising and segmentation of ECG signals.

#### 2.1.1. Noise Removal

**Figure 1:**
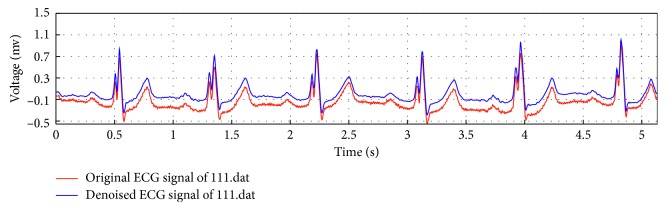
ECG signal denoised by db6 wavelet.

We denoised the raw data with the Daubechies 6 (db6) discrete wavelet transform [26], and the denoised ECG signals were input to the LSTM network. The original and denoised ECG signals are shown in Figure 1.

#### 2.1.2. ECG Beat Segmentation

**Figure 2:**
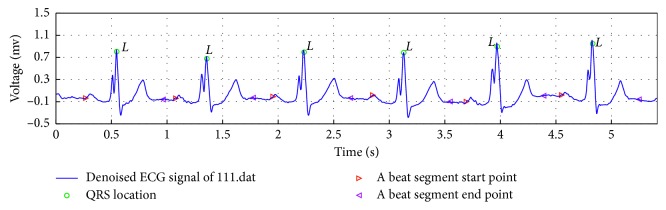
Sliding window search method extracts ECG beats.

We used the sliding window search method on the sample map extraction (see Figure 2). The MIT-BIH arrhythmia database provided annotations for ECG beat class information verified by independent experts. Since R-peak detection algorithms achieved more than 99% specificity and sensitivity [27–29], we used the R-peak annotation file directly. All ECG signals were segmented into sequences that were 250 samples long and centered on the annotated R-peaks. Note that we used an ECG beat with a length of 250 points by default, but there is no common standard for their size.

### 2.2. Method

#### 2.2.1. Problem Description

To achieve the detection of arrhythmia, the softmax regression model is used as the last layer of the LSTM network structure. For the input training set, *ℜ*={(*x*^(1)^, *y*^(1)^),…, (*x*^(*i*)^, *y*^(*i*)^),…, (*x*^(*n*)^, *y*^(*n*)^)}. *n* is the number of ECG beats containing the class labels. *x*^(*i*)^ is an ECG beat. *y*^(*i*)^ ∈ {0,1,2,3,4,5,6,7} is the category label of the *x*^(*i*)^. 0, 1, 2, 3, 4, 5, 6, and 7 are the representations of N, LBBB, RBBB, APC, NESC, ABERR, NPC, and AESC, respectively. If *y*=0, *x*^(*i*)^ is a N (normal); otherwise, *x*^(*i*)^ is one of the arrhythmia types. For an ECG beat *x*^(*i*)^, the output through the LSTM network is *z*^(*i*)^, as shown in

(1) $$\begin{array}{c} z^{\left(i\right)}=g\left(x^{\left(i\right)};\theta \right), \end{array}$$

where *g*(·) is a process function, describing the process of an ECG signal from the input layer to the last full connection layer, and *θ* is the relevant parameter in the LSTM network.

The last output vector *z*^(*i*)^ of full connection layer is ECG signal feature extracted by LSTM network. It is fed to the softmax layer which calculates the probability of each ECG beat category. Equation (2) is the softmax function used in the proposed network:

(2) $$\begin{array}{c} {\hat{y}}^{\left(i\right)}=\frac{\exp\left(z^{\left(i\right)}\right)}{\sum_{j=1}^{C}\exp\left(z^{\left(j\right)}\right)}, \end{array}$$

where *C* is the number of ECG beat categories. ${\hat{y}}^{\left(i\right)}$ is the class probability that the LSTM gives to the input feature vector *z*^(*i*)^.

#### 2.2.2. LSTM Recurrent Network

**Figure 3:**
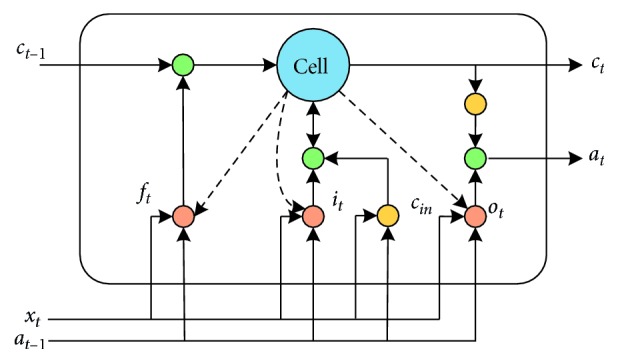
Long short-term memory block.

Long short-term memory (LSTM) is a time-recurrent neural network. It is suitable for time-series prediction of important events, and the delay interval is relatively long [30]. The neural network can effectively retain historical information and realize learning of long-term dependence information of text. The LSTM network consists of an input gate, forget gate, output gate, and cell unit to update and retain historical information.  Figure 3 shows an LSTM block.

The forget gate *f*_*t*_ in the LSTM memory block is controlled by a simple single neuron. It determines which information must be retained or discarded to enable the storage of historical information. The input gate *i*_*t*_ is a section where the LSTM block is created by a neuron and previous memory unit effects. It is activated to determine whether to update the historical information to the LSTM block. The candidate update content *c*_in_ is calculated by a tanh neuron. The current time memory cell state value *c*_*t*_ is calculated from the current candidate cell *c*_in_, the previous time state *c*_*t*−1_, the input gate information *i*_*t*_, and the forget gate information *f*_*t*_. *o*_*t*_ of the LSTM block at the current time is generated at the output gate. Finally, *a*_*t*_ determines the amount of information about the current cell state that will be output. The activation of each gate and the update of the current cell state can be calculated as follows:

(3) $$\begin{array}{c} f_{t}=\text{sigmoid}\left(W_{f}\cdot \left[a_{t-1},x_{t},c_{t-1}\right]+b_{f}\right),\\ i_{t}=\text{sigmoid}\left(W_{i}\cdot \left[a_{t-1},x_{t},c_{t-1}\right]+b_{i}\right),\\ c_{\text{in}}=\tanh\left(W_{c}\cdot \left[a_{t-1},x_{t},c_{t-1}\right]+b_{c}\right),\\ c_{t}=f_{t}\cdot c_{t}+i_{t}\cdot c_{\text{in}},\\ o_{t}=\text{sigmoid}\left(W_{o}\cdot \left[a_{t-1},x_{t},c_{t-1}\right]+b_{o}\right),\\ a_{t}=o_{t}\cdot \;\tanh\left(c_{t}\right). \end{array}$$

After calculating the hidden vector for each position, we considered the last hidden vector as the ECG signal representation. We fed it to a linear layer with an output length of the classification number and added a softmax output layer to classify the ECG beat as N, LBBB, RBBB, APC, NESC, ABERR, NPC, or AESC.

**Figure 4:**
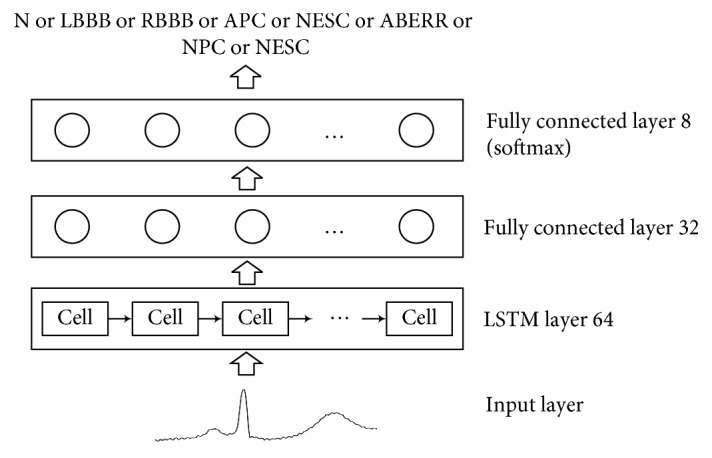
LSTM recurrent network architecture.

In this paper, we use the four-layer LSTM architecture including an input layer, an LSTM layer, and two fully connected layers. The structure of the proposed LSTM for imbalanced ECG signal feature extraction and classification tasks is shown in Figure 4.

#### 2.2.3. Focal Loss for Imbalanced ECG Beat Data

Focal loss is a more effective way to deal with the issue of imbalanced datasets. It is obtained by transforming the cross-entropy (CE) loss function. The CE is calculated by

(4) $$\begin{array}{c} \text{CE}\left(\hat{y}\right)=-\log\left(\hat{y}\right). \end{array}$$

**Figure 5:**
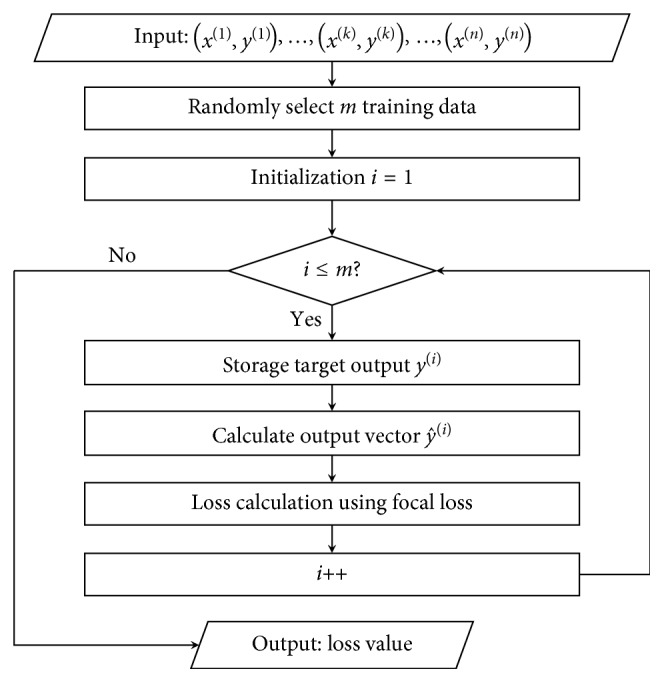
Block diagram to calculate loss value using focal loss.

The focal loss [31] is a dynamically scaled CE, where the scaling factor decays to zero as the confidence of the classification increases. Intuitively, this scaling factor can automatically downweight the contribution of normal ECG examples during training, and model training focuses quickly on the hard examples. The FL can be calculated by

(5) $$\begin{array}{c} \text{FL}\left(\hat{y}\right)=-{\left(1-\hat{y}\right)}^{\gamma }\cdot \;\log\left(\hat{y}\right),\;\gamma \geq 0, \end{array}$$

where ${\left(1-\hat{y}\right)}^{\gamma }$ is a modulating factor and *γ* is a focusing parameter. The purpose of the modulation factor is to reduce the weights of easily categorizable ECG beats so that the model is more focused on ECG beats that are difficult to classify during training. When an ECG beat is misclassified and $\hat{y}$ is small, the value of the modulation factor is close to 1 and the loss is barely affected. Loss value is calculated using FL according to the block diagram in Figure 5.

Optimization of the network parameters is important. There are many types of gradient descent optimization algorithms, such as Adagrad, Adadelta, Adam, and Nadam. This work uses the Nadam algorithm. This is an effective gradient descent optimization algorithm that combines the Adam and NAG algorithms to calculate adaptive learning rates for different parameters. Overall, Nadam performs better than other gradient descent optimization methods in practical applications [32].

## 3. Experiment and Results

### 3.1. Experiment Setup

**Figure 6:**
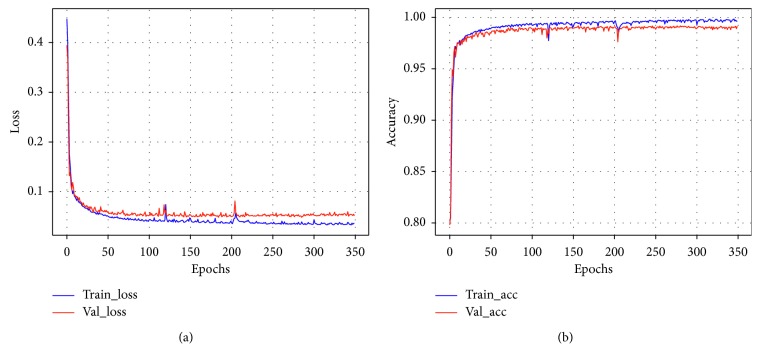
Training of the LSTM with FL (*γ*=2). (a) Loss curve. (b) Accuracy curve.

The LSTM network proposed in this study ran on the deep learning framework Tensorflow 1.12.0 in the Microsoft Windows 10 64 bit operating system. The computer server was configured with an 8-GB Intel (8) Core (TM) i5-7000 processor. Considering the effectiveness of the classification results, we set the epochs to 350. The loss curve and accuracy curve during the training and verification process of the LSTM network using FL (*γ*=2) are shown in Figure 6. By observing the curve of Figure 6, after 350 epochs, the network converged and the overall classification accuracy was stable. The average time required to train the model in one epoch was approximately 191 s. Please note that this epoch setting was only used to easily evaluate the impact of other learning parameters on the network classification results and is not guaranteed to be the best configuration for LSTM network.

### 3.2. Materials

We used the MIT-BIH arrhythmia database provided by the Massachusetts Institute of Technology [33]. It comes from 47 clinical patients and contains 48 annotated ECG records. Each group is approximately 30 minutes long and is sampled at a rate of 360 Hz by a 0.1–100 Hz band pass filter, for a total of approximately 650,000 sample points.

There are more than 109,000 marker beats from 16 heartbeat categories. All beats are marked by two or more cardiologists. The normal category has the most data volume, and the category with the least data are supraventricular premature beats (only two samples). This study used eight ECG beat types: N, LBBB, RBBB, APC, NESC, ABERR, NPC, and AESC. These beat types and their statistics are listed in Table 1.

From Table 1, it is found that there is a heavy imbalance between normal and abnormal ECG beats. Because of imbalanced ECG beat data, the network model tends to learn the distribution of major ECG beat data, while there is insufficient learning of minority ECG beat data, and we are often concerned with the lesser categories of abnormal ECG beats.

The dataset had a total of 93,371 ECG beats. We used 10% of all ECG data as the testing set. In the remaining ECG data, 90% of the data were used as the training set and 10% as the validation set. The training and validation sets were used to adjust the parameters and determine the optimal number of elements of the designed model. The model performance was evaluated using a testing set that was not previously used.

### 3.3. Evaluation Metrics

We used five metrics to evaluate the performance of the proposed network: accuracy, recall, precision, specificity, and *F*1 score. Accuracy is the proportion of correctly classified ECG beats of all ECG beats, which reflects the consistency between test results and real results. However, recall, precision, and specificity are less biased in evaluating the performance of the classifier on the imbalanced dataset. The *F*1 score is the harmonic mean of precision and recall. Five evaluation metrics can be calculated as follows:

(6) $$\begin{array}{c} \text{ACC}\left(\text{accuracy}\right)=\frac{\text{TPs}+\text{TNs}}{\text{TPs}+\text{TNs}+\text{FPs}+\text{FNs}},\\ \text{RE}\left(\text{recall}\right)=\frac{\text{TPs}}{\text{TPs}+\text{FNs}},\\ \text{PR}\left(\text{precision}\right)=\frac{\text{TPs}}{\text{TPs}+\text{FPs}},\\ \text{SP}\left(\text{specificity}\right)=\frac{\text{TNs}}{\text{TNs}+\text{FPs}},\\ F1=\frac{2\times \text{RE}\times \text{PR}}{\text{RE}+\text{PR}}. \end{array}$$

The classification categories in this study are not binary, so we use the confusion matrix to express the TP, FP, TN, and FN metrics built for a classification test. The confusion matrix makes it easy to generate the above four metrics.

### 3.4. Network Parameter Configuration

To obtain the best learning parameters of our proposed LSTM network, we quantitatively analyzed the impact of different learning parameters on the experimental results. The optimal parameter value was determined by evaluating the classification accuracy of the experimental results of multiple cases on the testing set.

After 350 epochs, the LSTM network converged and the classification accuracy was stable. The settings of the LSTM network parameters to obtain the best classification accuracy are shown in Table 2.

In this experiment, we analyzed the impact of various learning parameters on the classification performance of the proposed LSTM network with FL. The primary network parameters included the dropout, batch size, and *γ* parameter of FL.

We evaluated different dropouts for the proposed network with an increasing dropout proportion. The other learning parameter settings took the default values in Table 2.  Table 3 shows the classification accuracy on the testing set with different dropout proportions after 350 epochs.

By comparing the results of Table 3, we can see that the performance of our proposed LSTM network is not improved by increasing the dropout proportion. Therefore, the optimal dropout value of the LSTM network structure is around zero.

Then, we studied the effect on the LSTM network performance of changing the initial settings of the batch size. We evaluated the performance of five different batch sizes, as shown in Table 4.

Based on the results of Table 4, increasing or decreasing the size of the batch does not necessarily improve the performance of our proposed LSTM network. A larger batch size allows for more accurate estimation of the gradient, but it is prone to overfitting. The small batch size has a standardizing effect, but there is a risk of inefficiency, and it is not possible to stop or to not match the strategy early. For the dataset and network structure used in this paper, the optimal batch size is 128.

The *γ* parameter is the most critical parameter for FL. The effect of changing *γ* on the performance of our proposed LSTM network was investigated. The effect on the distribution of the loss for abnormal ECG beats was minor. For normal beats, however, increasing the value of the parameter *γ* heavily reduced the loss of correctly classified normal beats, allowing the model to focus on the misclassified abnormal ECG beats. After 350 epochs, the classification accuracy associated with the testing dataset was calculated and is given in Table 5 for six *γ* values. The other learning parameters are the same as in Table 2.

From the results shown in Table 5, we can see that increasing or decreasing *γ* did not improve the performance of the LSTM network with FL. The best *γ* parameter value is 2 for the proposed network.

## 4. Results and Discussion

In this study, we proposed a LSTM network structure to achieve the goal of imbalanced ECG signal classification. The ECG beat data were classified by the LSTM network, and then, we trained the LSTM network using FL. By setting the CE as the benchmark, the feasibility of using the FL to classify the imbalanced ECG beats was proved. We verified the effectiveness of the LSTM network structure by comparing with state-of-the-art methods.

**Figure 7:**
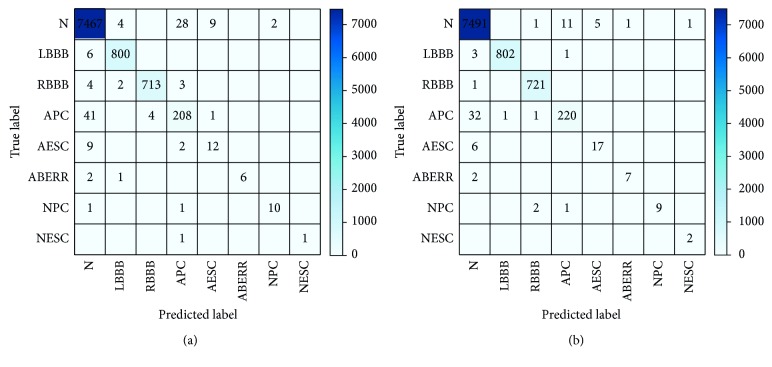
Confusion matrix obtained by the LSTM network using different loss functions on the testing set: (a) CE and (b) FL (*γ*=2).

Performance measures of the model were evaluated using a confusion matrix. The cost function of the LSTM network uses CE to calculate the confusion matrix on the testing set, as shown in Figure 7(a). The diagonal values in the confusion matrix represent the correct classification of ECG beats. Other LSTM network structure parameters (except *γ*) are the same as in Table 2. The cost function of the LSTM network uses FL to calculate the confusion matrix on the testing set, as shown in Figure 7(b). Other LSTM network parameters are the same as in Table 2.

By comparing and analyzing the confusion matrix of LSTM network with CE and LSTM network with FL in Figure 7, we can see that the LSTM network with FL performs better on the imbalanced ECG dataset than the LSTM network with CE. When the FL is examined, it appears that the LSTM network provides better recognition performance over most classes. Examining the CE, the LSTM network appears to provide lower recognition performance over most class. Also, for the CE, 41 APC beats are misclassified into *N* beats, while for the FL (*γ*=2), 32 APC beats are misclassified into *N* beats. This is because there is no big difference in the shape of the two beats, but there is a specific, difficult-to-position wave anomaly (e.g., the PR segment is extended).  Table 6 shows the PR, RE, SP, and *F*1 of the LSTM network with CE and LSTM network with FL on the testing set.

By comparing the results in Table 6, the validity of the LSTM network with FL is verified on imbalanced ECG data. From this table, it can be observed that the LSTM network with FL (*γ*=2) achieves an ACC of 99.26%, a RE of 99.26%, a PR of 99.30%, a SP of 99.14%, and an F1 score of 99.27%. The LSTM network with CE achieves 98.70% ACC, 98.70% RE, 98.05% PR, a SP of 98.75%, and 98.36% *F*1 score. Although the performance improvement by the LSTM network with FL seems not to be large compared to that of the LSTM network with CE, in a real diagnosis, even a minor accuracy improvement can hold great value for human health and life.

**Figure 8:**
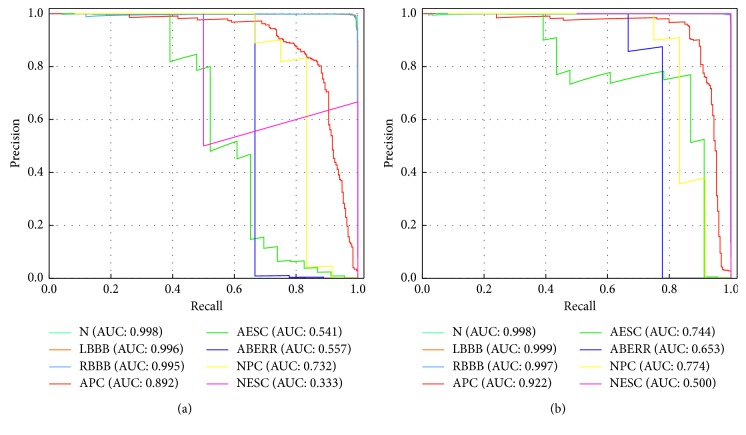
Precision-recall curves for every class. (a) Using CE and (b) using FL (*γ*=2) method.

To more intuitively compare the effectiveness of the above two methods (CE and FL), we next analyze the results using the precision-recall curve (PR curve). For the category imbalance problem, the PR curve is considered to be superior to the receiver operating characteristic curve (ROC curve) [34]. As shown in Figure 8, for the input of the imbalanced ECG data, the PR curve of each category is drawn from the classification results using the CE (shown in Figure 8(a)) and the FL (shown in Figure 8(b)), respectively. Compared with the CE, when the LSTM network proposed in this paper uses the FL, most categories obtain a relatively high area under the PR curve (AUC). Therefore, our proposed LSTM network with FL is effective in solving the category imbalance ECG dataset.

To verify the robustness of the proposed LSTM network with FL in a noisy environment, the network is also analyzed without denoised and the results are listed in Table 7. The performance measurements in Table 7 show that the LSTM network with FL (*γ*=2) achieved a classification result close to the result of denoised ECG recordings. It shows the advantages of denoised network and also illustrates the robustness of the network.

The proposed network can be deployed in telemedicine scenarios. The ECG data of heart patients are collected through wearable devices and transmitted to the cloud via the Internet. Data analysis is carried out through the proposed model in this study to assist cardiologists into more accurately and objectively diagnose ECG signals.

The proposed model was primarily studied on the MIT-BIH arrhythmia database. According to the AAMI standards (ANSI/AAMI EC57: 1998), all the beats in the MIT-BIH arrhythmia database are grouped into five main classes. However, this is not always desirable. The type of arrhythmia can be judged by the specific ECG beat and the regularity of the beat type. Repeated APC beats can become dangerous arrhythmias such as atrial fibrillation when a patient has a potential structural heart problem. Bundled branch blocks impede the normal pathway of electrical impulses through the conduction system to the ventricles. This causes asynchronous ventricular contractions and heart function deterioration, which may lead to life-threatening situations.

To assess the performance of the proposed network, we compared it to some state-of-the-art methods in the literature. We record the performance of the proposed network model (in bold) and the recent representative techniques for ECG beat classification using the MIT-BIH arrhythmia database in Table 8.

From Table 8, it is evident that our proposed LSTM network with FL achieved good performance. The difference between our study and other studies in the literature is that we used deep learning to classify category-imbalanced ECG beat data. For the classification of class-imbalanced ECG arrhythmias, we proposed a LSTM network with FL. There are also studies in the literature on the classification of imbalanced ECG data [24, 25]. The main difference is that our study uses FL that modifies the loss function, which makes the LSTM network more focused on feature learning of abnormal ECG beats that are prone to misclassification and improves the accuracy of arrhythmia classification. Regarding the RE, our proposed LSTM network with FL achieved a best result on the testing set. This means that it has a smaller number of false negatives, i.e., abnormal ECG beats which are erroneously classified as normal ECG beats. Furthermore, this method avoids the problem of the effective information reduction caused by the undersampling method or the problem of the network training time increase caused by the oversampling method.

The highlights of our proposed network are as follows:

- Feature extraction and selection techniques are not needed
- Our important finding is that the proposed method can improve the classification accuracy rate of categories with arrhythmia
- Our proposed method is robust under without denoised ECG recordings

The disadvantages of our proposed network are as follows:

- This study is conducted only on eight ECG beat types
- The proposed network is the time cost of the training phase

## 5. Conclusions and Future Work

In this study, we proposed a LSTM network with FL to improve the training effect by inhibiting the impact of a large number of easy normal ECG beat data on model training. The results show that the LSTM network with FL achieved an accuracy, recall, precision, specificity, and *F*1 score of 99.26%, 99.26%, 99.30%, 99.14%, and 99.27%, respectively. Experimental results of the MIT-BIH arrhythmia database demonstrate the effectiveness and robustness of the proposed network. The proposed method can be deployed in telemedicine scenarios to assist cardiologists into more accurately and objectively diagnosing ECG signals.

The study was conducted only on eight ECG beat types. To generalize the results, various types and numerous beats should be incorporated in future research. And, we also plan to add different levels of noise to ECG signals to discuss the performance of the LSTM with the FL model.

## Figures and Tables

**Table 1:** ECG beat types used in this work.

ECG beat types	Annotation	Number of beats
Normal beat	N	75,020
Left bundle branch block	LBBB	8,072
Right bundle branch block	RBBB	7,255
Atrial premature contraction	APC	2,546
Nodal (junctional) escape beat	NESC	229
Aberrated atrial premature beat	ABERR	150
Nodal (junctional) premature beat	NPC	83
Atrial escape beat	AESC	16
Total	—	93,371

**Table 2:** LSTM network default parameter settings.

LSTM cells	Network layers	Optimizer	Dropout	Epoch	Batch size	Cost function	*γ*
64	4	Nadam	0	350	128	Focal loss	2

**Table 3:** Classification accuracy of different dropout proportions.

Dropout	0	0.10	0.15	0.20	0.25	0.30
ACC of testing set (%)	99.26	98.7	99.21	98.46	99.09	99.07

**Table 4:** Classification accuracy of different batch sizes.

Batch size	32	64	128	256	512
ACC of testing set (%)	81.20	97.87	99.26	99.11	98.72

**Table 5:** Overall accuracy of FL over different *γ* parameter.

*γ* parameter	0	0.5	1	2	3	4
ACC of testing set (%)	98.85	99.08	99.03	99.26	99.11	98.82

**Table 6:** LSTM network classification results on the testing set using two different loss methods.

Cost function	ACC (%)	RE (%)	SP (%)	PR (%)	*F*1 (%)
Cross entropy	98.70	98.70	98.05	98.75	98.36
Focal loss (*γ*=2)	99.26	99.26	99.14	99.30	99.27

**Table 7:** LSTM network with FL (*γ*=2) classification results on the testing set.

	ACC (%)	RE (%)	SP (%)	PR (%)	*F*1 (%)
Denoised	99.26	99.26	99.14	99.30	99.27
Without denoised	99.07	99.07	98.99	99.13	99.09

**Table 8:** Comparison between the related work and the method proposed in this work.

Works	Year	Classes	Methods	ACC (%)	RE (%)	SP (%)
Martis et al. [35]	2013	5 beat types	DCT + PCA, PNN	99.52	98.69	99.91
Raj et al. [7]	2016	16 beat types	DOST, SVM-PSO	99.18	—	—
Sharma and Ray [36]	2016	6 beat types	EMD, HHT, SVM	99.51	98.64	99.77
Gutiérrez-Gnecchi et al. [37]	2017	8 beat types	PNN	98.89	—	—
Jung and Lee [38]	2017	4 beat types	WKNN	96.12	96.12	99.97
Li et al. [6]	2017	6 beat types	GA-BPNN	97.78	97.86	99.54
Rajesh and Dhuli [25]	2018	5 beat groups	DBB, AdaBoost	99.10	97.90	99.40
W. Li and J. Li [14]	2018	16 beat types	LDP, DNN	98.37	—	—
Yildirim. [20]	2018	5 beat types	DULSTM-WS2	99.25	—	—
Oh et al. [22]	2018	5 beat types	CNN-LSTM	98.10	97.50	98.70
Pławiak and Acharya [39]	2019	17 classes	DGEC	99.37	94.62	99.66
Yildirim et al. [21]	2019	5 beat types	LSTM	99.23	99.00	99.00
**Our work**	**2019**	**8 beat types**	**LSTM, FL**	**99.26**	**99.26**	**99.14**

DCT: discrete cosine transform; GMM + EM: Gaussian mixture modeling with enhanced expectation maximization; DOST: discrete orthogonal stockwell transform; SVM-PSO: PSO-tuned support vector machine; EMD: empirical mode decomposition; HHT: Hilbert–Huang transform; PNN: probabilistic neural network; WKNN: weighted *k*-nearest neighbor; NRSC: neighborhood rough set; DWT: discrete wavelet transform; GA-BPNN: genetic algorithm-backpropagation neural network; DNN: deep neural network; DULSTM-WS: deep unidirectional LSTM network-based wavelet sequences; DBLSTM-WS: deep bidirectional LSTM network-based wavelet sequences; LDP: local deep field; DBB: distribution-based balancing; FL: focal loss; DGEC: deep genetic ensemble of classifiers.

## Data Availability

The data used to support the findings of this study are included in the article. Further data can be requested from the corresponding author.
